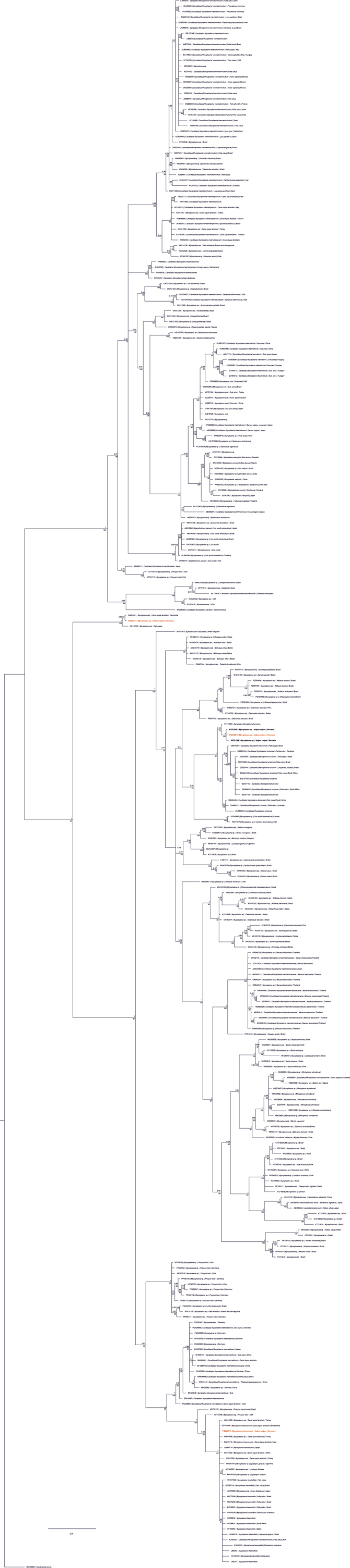# Molecular analysis of vector-borne pathogens in red foxes (*Vulpes vulpes*) from Saxony-Anhalt (Germany)

**DOI:** 10.1016/j.ijppaw.2025.101162

**Published:** 2025-11-18

**Authors:** Zoë Tess Lara Lindhorst, Manuela Theresa Frangl, Barbara Eigner, Bita Shahi Barogh, Georg Gerhard Duscher, Annette Schliephake, Wolfgang Gaede, Hans-Peter Fuehrer, Mike Heddergott

**Affiliations:** a Institute of Parasitology, Department of Pathobiology, University of Veterinary Medicine Vienna, Austria; b Musée National d’Histoire Naturelle, Luxembourg; c Institute for Veterinary Disease Control Mödling, AGES – Austrian Agency for Health and Food Safety, Austria; d Department for Veterinary Medicine, State Institute for Consumer Protection of Saxony-Anhalt, Haferbreiter Weg 132-135, 39576, Stendal, Germany

**Keywords:** Wild carnivores, Bacteria, Protozoa, Arthropods, Acari, Insecta

## Abstract

Vector-borne pathogens (VBPs) are becoming increasingly important in veterinary medicine and public health, with wildlife potentially playing a key role in their transmission. The objective of the current study was to investigate the occurrence of vector-borne pathogens in red foxes (*Vulpes vulpes*). Spleen samples from 277 legally hunted foxes were collected over a period of twelve months (May 2020 to April 2021) in Saxony-Anhalt, Germany. VBPs were identified by performing PCR analysis on the samples, followed by Sanger sequencing, and a phylogenetic analysis was performed on *Mycoplasma* spp. A total of 94 % of the samples showed a positive result. The pathogens identified were *Hepatozoon* spp. (77 %), *Babesia vulpes* (68 %), *Mycoplasma haemocanis* (5 %), *Mycoplasma* spp. (5 %), *Bartonella taylorii* (1 %), *Bartonella rochalimae* (0.7 %), and *Trypanosoma pestanai* (0.4 %). None of the examined samples tested positive for filarioid helminths, *Rickettsia* spp., and Anaplasmataceae. This study highlights the role of foxes as reservoirs for pathogens that may affect domestic animals and humans, potentially contributing to the spread of these pathogens through shared environments and vectors.

## Highlights

• A very high occurrence of vector-borne pathogens was found (95 %).
• The most frequently detected pathogens were *H. canis* (77 %) and *B. vulpes* (71 %).
• First report of *T. pestanai* globally among red foxes.
• First report of *B. taylorii* in red foxes and of *B. rochalimae* in Germany.

## Graphical abstract

**Figure:**
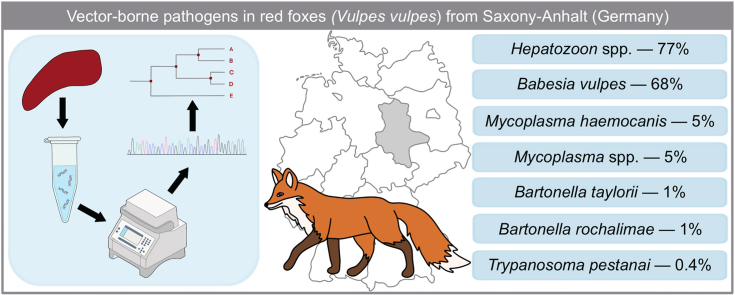

## 1 Introduction

The red fox (*Vulpes vulpes*), hereafter referred to simply as “fox”, is a medium-sized carnivore and the most widespread carnivore in the world (Lindsø et al., 2022). Its original range covers a large part of the northern hemisphere. Populations that can be traced back to introductions also exist in parts of North America and Australia (Soe et al., 2017). This predator inhabits a wide range of biotopes, including wetlands, grasslands, deciduous and coniferous woodlands, as well as riparian areas. Foxes have high ecological plasticity and have adapted to different habitats in recent decades (Kimmig et al., 2020). In the middle of the last century, rabies first appeared among foxes in Europe (Adamson, 1977), having a negative impact on the population. However, with the beginning of the successful control of rabies through oral vaccination in many parts of Europe, the population began to grow rapidly. One consequence of this population growth in recent decades has been the increasing colonization of urban and suburban areas (Deplazes et al., 2004), which can inevitably lead to contact with humans and domestic animals (Sakalauskas et al., 2019). Due to the high number of foxes, including many living in close proximity to urban areas, and the increased presence of wildlife near humans observed during the COVID-19 pandemic (2020–2023) (Moesch et al., 2024), fox populations could pose a significant threat to the health of humans, domestic animals, and wildlife through the transmission of viral, bacterial, and parasitic pathogens (Kosoy and Goodrich, 2019; Lempp et al., 2017).

Red foxes host several pathogens, including endo- and ectoparasites (Waindok et al., 2021) as well as vector-borne pathogens (VBPs) (Duscher et al., 2015). Among endoparasites, *Echinococcus multilocularis* (the fox tapeworm) is particularly significant due to its extensive distribution and pronounced zoonotic potential (Conraths and Maksimov, 2020). Among zoonotic VBPs, the heartworm (*Dirofilaria immitis*) and skin worm (*Dirofilaria repens*) are of particular interest, having been reported in foxes across several European countries (Cirović et al., 2014; Fuehrer et al., 2021; Ionică et al., 2017; Magi et al., 2009; Medkour et al., 2020; Penezić et al., 2014; Potkonjak et al., 2020; Tolnai et al., 2014). While foxes are potential reservoirs for these parasites (Magi et al., 2008), to the authors’ knowledge, zoonotic filariae have not yet been detected in foxes in Germany (Härtwig et al., 2016).

Apicomplexan parasites widely spread among European fox populations are *Babesia vulpes* (Baneth et al., 2015, 2019) (formerly known as *Theileria annae, Babesia* sp. “Spanish dog”*,* or *Babesia* cf. *microti*) and *Hepatozoon canis* (Duscher et al., 2015; Hodžić et al., 2018; Koneval et al., 2017; Lesiczka et al., 2023; Liesner et al., 2016; Medkour et al., 2020; Víchová et al., 2018), both of which can also infect other canids, including domestic dogs (*Canis lupus familiaris*), potentially causing severe symptoms (Barash et al., 2019; Unterköfler et al., 2023; Vincent-Johnson et al., 1997).

*Bartonella rochalimae*, the most prevalent *Bartonella* species detected in foxes in Europe (Gerrikagoitia et al., 2012; Greco et al., 2021; Henn et al., 2009a; Hodžić et al., 2018; Millán et al., 2016), is a vector-borne bacterium with potential to infect humans (Eremeeva et al., 2007) and other carnivores, occasionally causing endocarditis (Henn et al., 2009b). *Anaplasma phagocytophilum*, the causative agent of human granulocytic anaplasmosis, as well as canine and equine anaplasmosis, and tick-borne fever in ruminants (Woldehiwet, 2010), was detected in foxes from multiple European countries (Dumitrache et al., 2015; Ebani et al., 2011; Härtwig et al., 2014; Hodžić et al., 2018; Hofmann-Lehmann et al., 2016; Jahfari et al., 2014; Karbowiak et al., 2009; Lesiczka et al., 2023; Tolnai et al., 2015). Other pathogens in the order Rickettsiales detected in foxes in Europe include *Anaplasma platys* (Cardoso et al., 2015; Medkour et al., 2020), *Ehrlichia canis* (Cardoso et al., 2015; Ebani et al., 2017; Millán et al., 2016; Santoro et al., 2016; Sgroi et al., 2021b; Torina et al., 2013), *Candidatus* Neoehrlichia spp. (Hodžić et al., 2017a, 2018; Lesiczka et al., 2023), and *Rickettsia* spp. (Hofmann-Lehmann et al., 2016; Lledó et al., 2016; Ortuño et al., 2018). Hemotropic mycoplasmas, also known as hemoplasmas can cause acute hemolytic anemia and various chronic diseases, and can infect different vertebrate hosts (Messick, 2004), including humans (Steer et al., 2011) and carnivores, such as foxes (Millán et al., 2018).

The objective of our research was to investigate the occurrence of Anaplasmataceae, *Bartonella* spp., hemoplasmas, *Rickettsia* spp., Piroplasmida, Trypanosomatidae and Filarioidea in foxes in Saxony-Anhalt, in order to enhance understanding of their epidemiology and to support the assessment of potential transmission risks to humans, domestic animals, and wildlife.

## 2 Materials and methods

### 2.1 Sample collection

**Fig. 1:**
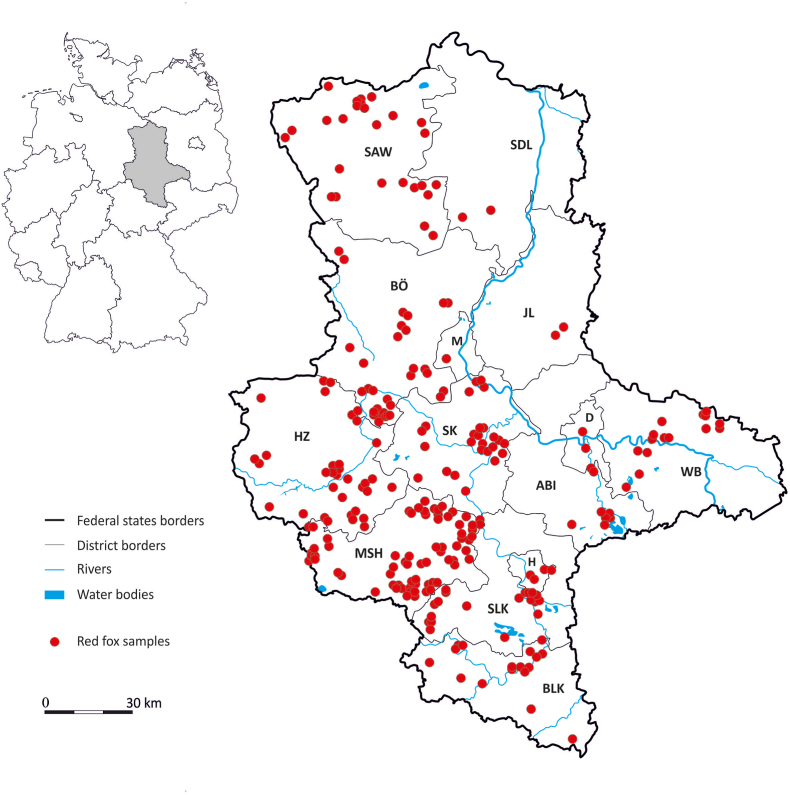
Geographic origin of the 277 red fox (*Vulpes vulpes*) in Saxony-Anhalt included in this study. Abbreviations: Altmarkkreis Salzwedel (SAW), Anhalt-Bitterfeld (ABI), Burgenlandkreis (BLK), Börde (BÖ), Harz (HZ), Jerichower Land (JL), Mansfeld-Südharz (MSH), Saalekreis (SKA), Salzlandkreis (SLK), Stendal (SDL), Wittenberg (WB), City of Halle (Saale) (H), City of Dessau-Roßlau (D) and City of Magdeburg (M).

Spleen samples were collected over a twelve-month period (May 2020 to April 2021) from 277 foxes found dead or legally shot in Saxony-Anhalt (Fig. 1). Of these, 140 samples were collected in 2020 and 137 in 2021. The foxes originated from 11 different districts and 3 independent cities (Table 1, Fig. 1). Carcasses were typically submitted within a week of being found or hunted. Following necropsy at the Department of Veterinary Medicine of the Saxony-Anhalt State Office of Consumer Protection for Rabies virus (RABV) testing, spleen samples were preserved in 70 % ethanol until further analysis at the University of Veterinary Medicine, Vienna. During necropsy, each fox was examined to determine sex and age based on dentition stage and tooth attrition (Habermehl, 1985), classifying individuals as either adults (>1 year) or juveniles (≤1 year).

**Table 1:** Origin, sex, and age of red foxes (*Vulpes vulpes*) presented in the present study, N = 277.

District/city	Male		Female	
	Adult	Juvenile	Adult	Juvenile
Altmarkkreis Salzwedel	14	3	14	1
Anhalt-Bitterfeld	5	1	2	–
Burgenlandkreis	14	–	4	–
Börde	13	–	15	1
Harz	14	3	15	3
Jerichower Land	1	–	1	–
Mansfeld-Südharz	48	1	30	3
Saalekreis	12	1	7	1
Salzlandkreis	15	–	7	2
Stendal	1	–	1	–
Wittenberg	6	1	5	2
City of Halle (Saale)	3	1	2	1
City of Dessau-Roßlau	2	–	–	–
City of Magdeburg	1	–	–	–
Total	11	149	14	103

### 2.2 DNA extraction, PCR amplification, and sequencing

DNA was isolated from spleen samples using the DNeasy Blood & Tissue kit (250) (QIAGEN, Hilden, Germany), according to the manufacturer's instructions. The pathogens were detected using seven different established broad-range PCR protocols (Table 2, Supplementary File 1, Table S1), including a positive and a negative control.

**Table 2:** Oligonucleotide sequences of primers used in the present study.

Target organism (genetic marker)	Primer sequences (5′→3′)	Product size	Reference
*Mycoplasma* spp. (16S rRNA)	HBT-F: ATA CGG CCC ATA TTC CTA CG	600 bp	Criado-Fornelio et al. (2003a)
	HBT-R: TGC TCC ACC ACT TGT TCA		
Piroplasmida (18S rRNA)	BTH-1F: CCT GAG AAA CGG CTA CCA CAT CT	700 bp	Bonnet et al., (2007); Criado-Fornelio et al., (2003b)
	BTH-1R: TTG CGA CCA TAC TCC CCC CA		
	G-2_for: GTC TTG TAA TTG GAA TGA TGG	561 bp	
	G-2_rev: CCA AAG ACT TTG ATT TCT CTC		
Trypanosomatida (18S rRNA)	Tryp_18S_F: GTG GAC TGC CAT GGC GTT GA	1320 bp	Peña-Espinoza et al. (2023)
	Tryp_18S_R: CAG CTT GGA TCT CGT CCG TTG A		
	Tryp_18S_F2: CGA TGA GGC AGC GAA AAG AAA TAG AG	960 bp	
	Tryp_18S_R2: GAC TGT AAC CTC AAA GCT TTC GCG		
*Bartonella* spp. (*gltA*)	BhCS.781p: GGG GAC CAG CTC ATG GTG G	179 bp	Norman et al. (1995)
	BhCS.1137n: AAT GCA AAA AGA ACA GTA AAC A		
*Rickettsia* spp. (23S-5S rRNA)	Ricketts_ITS_for: GAT AGG TCG GGT GTG GAA G	∼400 bp	Vitorino et al. (2003)
	Ricketts_ITS_rev: TCG GGA TGG GAT CGT GTG		
Anaplasmataceae (16S rRNA)	EHR16SD_for: GGT ACC YAC AGA AGA AGT CC	345 bp	Parola et al. (2000)
	EHR16SR_rev: TAG CAC TCA TCG TTT ACA GC		
Filarioidea (*COI*)	COIint-F: TGA TTG GTG GTT TTG GTA A	668 bp	Casiraghi et al. (2001)
	COIint-R: ATA AGT ACG AGT ATC AAT ATC		
Hepatozoon spp. (18S rRNA)	H14Hepa18SFw: GAA ATA ACA ATA CAA GGC AGT TAA AAT GCT	∼620 bp	Hodžić et al. (2015)
	H14Hepa18SRv: GTG CTG AAG GAG TCG TTT ATA AAG A		

The PCR assays targeted sections of the *16S* ribosomal RNA (rRNA) in *Mycoplasma* spp. and Anaplasmataceae, the *18S* rRNA in Piroplasmida, *Hepatozoon* spp., and Trypanosomatidae, the citrate synthase gene *(gltA)* in *Bartonella* spp., the *23S-5S* rRNA intergenic spacer in *Rickettsia* spp. and the cytochrome *c* oxidase subunit 1 mitochondrial gene (*COI*) in Filarioidea.

PCRs were run using GoTaq™ DNA Polymerase (Promega, Madison, WI, USA) under the conditions specified by the manufacturer (Supplementary File 1, Table S1). Following the PCR, gel electrophoresis (48 min at 120V) was conducted, using 1.8 % agarose gel with Midori Green Advance DNA Stain (NIPPON Genetics, Germany). Subsequently, the positive samples were sent to LGC Genomics (Berlin, Germany) for Sanger sequencing. The chromatograms were visually inspected and edited (using BioEdit v.7.2.5 (Hall, 1999)) and compared to sequences deposited in the NCBI GenBank using BLAST analysis.

### 2.3 Phylogenetic analysis

One *Mycoplasma* sequence obtained in the present study (GenBank accession no.: **PQ834270**) was used to search for similar sequences using the BLAST function on NCBI GenBank, setting the number of maximum target sequences to 5000. The filter was set to 79–100 % identity and 98–100 % query coverage. The sequences were aligned and sorted using the default option (FFT–NS–2) in MAFFT v.7.520 (Katoh and Standley, 2013). All sequences featuring obvious sequencing errors and ambiguous characters were removed from the alignment and excluded from the analysis.

To provide an overview of the diversity of haplotypes, maximum likelihood (ML) and Bayesian inference (BI) trees were calculated based on an alignment containing 773 sequences (623 nucleotide positions). Gaps were removed from the alignment using TrimAl v.1.3 (http://phylemon2.bioinfo.cipf.es/ (Sánchez et al., 2011);), and sequences were collapsed to haplotypes using DAMBE v.7.3.32, leaving 289 haplotypes (549 nucleotide positions). The tree was rooted with a sequence of *Brevibacillus brevis* (GenBank accession no.: **MG456833**).

An ML bootstrap consensus tree (1000 replicates) was calculated using the W-IQ-TREE web server (http://iqtree.cibiv.univie.ac.at*/* (Trifinopoulos et al., 2016);*)* applying the model TIM3 + I + G4, which was suggested as the best fit for the data set in the model test according to the Bayesian inference criterion (BIC). The BI trees were calculated using MrBayes v.3.2.7 (Ronquist et al., 2012), applying the model GTR + G + I. The analysis was run for 10^6^ generations (2 runs each with 4 chains), sampling every thousandth tree. The first 25 % of trees were discarded as burn-in and a 50 % majority-rule consensus tree was calculated based on the remaining 750 trees. The ML and BI trees were jointly created using the BI tree as a template, and then graphically prepared indicating country and host information in CorelDRAW 2024 (Corel, Ottawa, ON, Canada).

For reasons of clarity, a reduced tree was created with the number of sequences being reduced to up to 4 sequences per clade, leaving 38 sequences (549 nucleotide positions).

### 2.4 Statistical analysis

Statistical analysis was conducted using R version 4.3.2 ® (Foundation for Statistical Computing, Vienna, Austria) to detect correlation between pathogen occurrence and sex, age, year, month, and geographic location (administrative district) of the foxes' death. A Pearson's Chi-squared (*χ*2) test and a Fisher's exact test were conducted. Due to computational limitations encountered during the Fisher's exact test caused by the dataset's complexity, a Monte Carlo simulation was used in some cases to estimate *p*-values. Effects were considered statistically significant if *P* < 0.05. To determine the 95 % confidence intervals (CIs) for the proportions, the Clopper-Pearson exact method was employed.

In the case of *Hepatozoon*, for statistical analyses, all positive samples were treated as *H*. *canis*. This decision was based on the sequencing results and the absence of reports of other *Hepatozoon* species in foxes in Europe.

## 3 Results

In total, 259/277 (93.5 %; 95 % CI 0.902–0.961) spleen samples tested positive for one or more pathogens, while the remaining 15/277 (6.5 %; 95 % CI 0.039–0.098) showed negative results for all tested pathogens. The most frequently detected pathogen among the foxes was *Hepatozoon* spp. (212/277, 76.5 %; 95 % CI 0.710–0.814). Other pathogens detected were *B. vulpes* (188/277, 67.9 %; 95 % CI 0.622–0.731), *Mycoplasma haemocanis (Mhc)* (14/277, 5.1 %; 95 % CI 0.028–0.083), *Mycoplasma* spp. (13/277, 4.7 %; 95 % CI 0.025–0.078), *Bartonella taylorii* (3/277, 1.1 %; 95 % CI 0.002–0.031), *Bartonella rochalimae* (2/277, 0.7 %; 95 % CI 0.001–0.024), and *Trypanosoma pestanai* (1/277, 0.4 %; 95 % CI 0.000–0.021) (Table 3, Table 4). None of the examined samples tested positive for filarioid helminths, *Rickettsia* spp., and Anaplasmataceae.

**Table 3:** Molecular results and distribution of vector-borne pathogens surveyed in samples from red foxes (*Vulpes vulpes*) from Saxony-Anhalt, Germany, showing the prevalence of pathogens and their distribution according to fox sex and age (n/N (%)). *H* = *Hepatozoon*, *B. canis* = *Babesia canis, B. taylorii = Bartonella taylorii, B. rochalimae = Bartonella rochalimae*, *M* = *Mycoplasma*, *T* = *Trypanosoma*. ∗ Statistically significant correlation (*P* < 0.05),^1^ All sequenced samples were identified as *H. canis*. Unsequenced positives were formally designated as *Hepatozoon* spp., but for simplicity, prevalence is presented here as *H. canis*.

Pathogen		Sex			Age		
	Prevalence	Male	Female	*P*	Juvenile	Adult	*P*
*H. canis*^1^	212/277 (77 %)	116/160 (73 %)	96/117 (82 %)	0.090	25/25 (100 %)	187/252 (74 %)	0.002∗
*B. vulpes*	188/277 (71 %)	114/160 (71 %)	74/117 (63 %)	0.201	15/25 (60 %)	173/252 (69 %)	0.510
*M. haemocanis*	14/277 (5 %)	9/160 (6 %)	5/117 (4 %)	0.595	0/25 (0 %)	14/252 (6 %)	0.398
*Mycoplasma* sp.	13/277 (5 %)	9/160 (6 %)	4/117 (3 %)		0/25 (0 %)	13/252 (5 %)	
*T. pestanai*	1/277 (0.4 %)	1/160 (0.6 %)	0/117 (0 %)	1	0/25 (0 %)	1/252 (0.4 %)	1
*B. taylorii*	3/277	1/160 (0.6 %)	2/117 (1.7 %)	0.500	0/25 (0 %)	2/252 (0.8 %)	0.379
*B. rochalimae*	2/277	2/160 (1.3 %)	0/117 (0 %)		1/25 (4 %)	2/252 (0.8 %)

**Table 4:** Sequencing results for pathogens found in red foxes (*Vulpes vulpes*) in Germany and their closest relationship based on GenBank BLAST results. Id = Identity, QC = Query Coverage, *B* = *Babesia*, *M* = *Mycoplasma*, *T* = *Trypanosoma*.^1^ Altogether, 212 fox samples tested positive for *Hepatozoon* by PCR; to confirm species identity, seven representative samples were sequenced.

Number of samples	Accession no. (this study)	Reference haplotype	Reference accession no.	Reference country	Reference host	Id (%)	QC (%)
7^1^	**PX308631- PX308633**	*H. canis*	**ON128264**	Czechia	*Vulpes vulpes*	100	100
188	**PQ827005**	*B. vulpes*	**KM115968**	Austria	*Vulpes vulpes*	100	100
14	**PQ834270**	*M. haemocanis*	**GQ129115**	Italy	*Canis lupus familiaris*	100	100
10	**PQ834271**	*Mycoplasma* sp.	**KX761385**	Slovakia	*Vulpes vulpes*	100	100
3	**PQ834272**	*Mycoplasma* sp.	**ON620261**	Cambodia	*Canis lupus familiaris*	100	100
1	**PQ826483**	*T. pestanai*	**PP595229**	Romania	*Meles meles*	99.8	98
3	**PX400655-PX400657**	*B. taylorii*	**PV170757**	Czechia	NA	100	100
2	**PX400658-PX400659**	*B. rochalimae*	**PV170758**	Czechia	NA	100	100

Among male and female foxes, 92.5 % (148/160; 95 % CI 0.876–0.963) and 94.9 % (111/117; 95 % CI 0.893–0.982), respectively, tested positive for one or more pathogens. Regarding the age of the animals, 92.9 % (234/252; 95 % CI 0.889–0.957) of adults and 100 % (25/25; 95 % CI 0.863–1.0) of juveniles showed an infection with at least one pathogen.

**Fig. 2:**
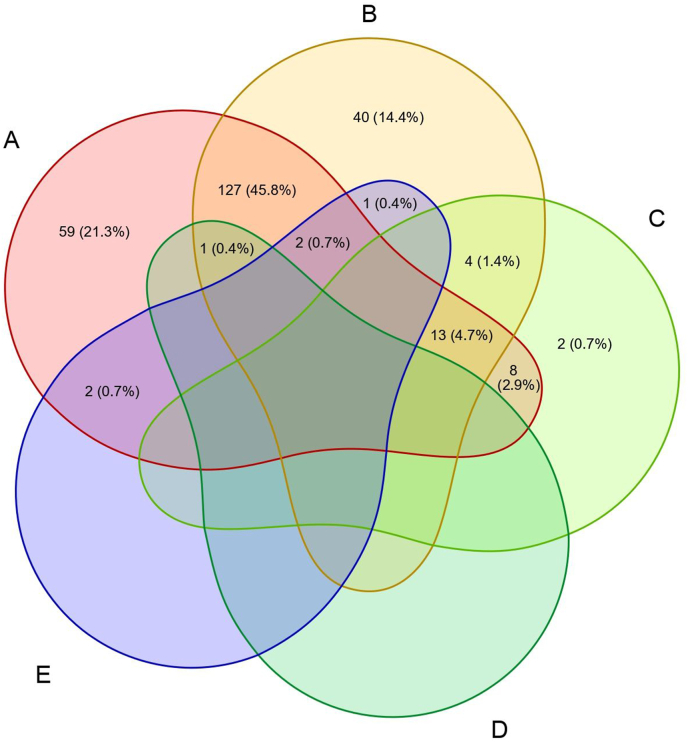
Co-infection scheme of detected pathogens. Numbers represent counts of red foxes (*Vulpes vulpes*) with respective pathogens detected. Percentages represent the proportion of positive foxes among all foxes tested (n = 277). **A**, *Hepatozoon canis*; **B**, *Babesia vulpes*; **C**, hemotropic mycoplasmas; **D**, *Trypanosoma pestanai*; **E**, *Bartonella* spp.

While 101/259 (39.0 %; 95 % CI 0.326–0.458) of the foxes were infected with one pathogen only, 142/259 (54.8 %; 95 % CI 0.484–0.610) and 16/259 (6.2 %; 95 % CI 0.036–0.098) showed a co-infection with two and three different pathogens, respectively (Fig. 2). No animal was infected with more than three pathogens.

**Fig. 3:**
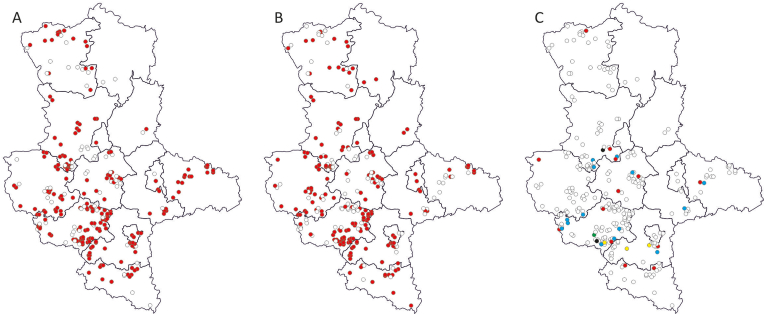
Geographical distribution of non-infected (white dots) and infected red foxes (*Vulpes vulpes*) from Saxony-Anhalt according to pathogens detected. **A:** red dots stand for the detection of *Hepatozoon* spp.; **B:** red dots stand for the detection of *Babesia vulpes*; **C:** red dots stand for the detection of *Mycoplasma* spp., blue dots stand for *Mycoplasma haemocanis*, black dots stand for *Bartonella rochalimae*, yellow dots stand for *Bartonella taylorii* and the green dot stands for *Trypanosoma pestanai*.

The districts of Anhalt-Bitterfeld, Harz, Jerichower Land, Saalekreis, Stendal, Wittenberg, as well as the cities of Dessau-Roßlau, Halle (Saale), and Magdeburg exhibited the highest prevalence of pathogens (100 % each). These were followed by Altmarkkreis Salzwedel (97 %), Mansfeld-Südharz (94 %), Burgenlandkreis (89 %), Börde (86 %), and Salzlandkreis (83 %) (Fig. 3A, B, and 3C). Nonetheless, no significant associations were identified between pathogen occurrence and any of the evaluated parameters, i.e. sampling year, month, location, sex, or age.

Similarly, when individual pathogens were analyzed, most parameters showed no significant correlation. However, a notable exception was observed with *Hepatozoon*, where a significant relationship was found with age (*P* = 0.002, odds ratio [OR] Infinite, 95 % CI 2.102-Infinity) (Table 3) and sampling location (*P* = 0.037). Among adult foxes, 74.2 % (187/252; 95 % CI: 0.682–0.795) tested positive for *Hepatozoon*, while all juveniles tested positive (100 %, 25/25; 95 % CI: 0.863–0.1) (Table 3). Regarding location-specific results, most districts had a higher proportion of infected animals compared to non-infected ones. In four districts, all animals tested were positive for *Hepoatozoon* (100 %; 1/1, 1/1, 2/2, and 14/14; 95 % CI 0.025–1, 0.158–1, and 0.768–1). Conversely, one district had an equal number of positive and negative samples (50 %, each 1/2; 95 % CI 0.013–0.987), while another district reported no positive cases at all (0/2; 95 % CI 0–0.842) (Fig. 3A).

The most frequently detected pathogen was *Hepatozoon* spp. Seven of the 212 PCR-positive samples were randomly selected and sent for sequencing as references, all of which showed 100 % identity and query coverage to *H. canis*, detected in a fox from Czech Republic (GenBank accession no. **ON128264**). Three sequences were uploaded to GenBank under accession nos. **PX308631**–**PX308633**). Given these results and the epidemiological context, it is highly likely that all positive samples represent *H. canis*. Nevertheless, in order to remain conservative, unsequenced samples are reported as *Hepatozoon* spp.

In total, 188 samples tested positive for *B. vulpes*. All obtained sequences were 100 % identical, showing no variation among samples. Therefore, one representative sequence was uploaded to GenBank under accession no. **PQ827005**, showing both 100 % identity and query coverage to *B. vulpes*, detected in a fox from Austria (GenBank accession no. **KM115968**) (Table 4).

Twenty-seven samples tested positive for hemoplasmas. Out of these, 14/27 (51.9 %; 95 % CI 0.319–0.713) were identical and showed 100 % identity and 100 % query coverage to *Mhc*, detected in a dog in Italy (GenBank accession no. **GQ129115**). One representative sequence has been deposited in GenBank under accession no. **PQ834270**. Additionally, 10/27 (37.0 %; 95 % CI 0.194–0.576) sequences showed highest identity to *Mycoplasma* sp. in a fox from Slovakia (GenBank accession no. **KX761385**; one representative sequence was uploaded to GenBank under accession no. **PQ834271**. Lastly, 3/27 (11.1 %; 95 % CI 0.024–0.292) sequences that were identical, showed 100 % identity and 100 % query coverage to *Mycoplasma* sp., detected in a dog from Cambodia (GenBank accession no. **ON620261**), and one representative sequence was submitted to GenBank under accession no. **PQ834272** (Table 4).

**Fig. 4:**
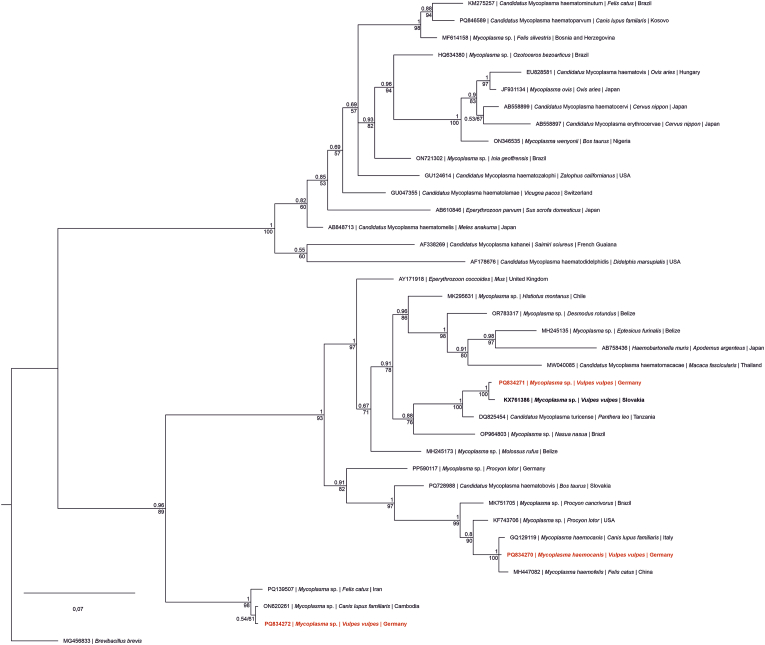
Bayesian Inference (BI) tree featuring 16S rRNA (549 nucleotide positions) sequences of *Mycoplasma* spp. Nodes are marked with Bayesian posterior probabilities and Maximum Likelihood (ML) bootstrap values. Accession number, species name, and host species are provided for every sequence. Sequences written in bold are from red fox (*Vulpes vulpes*), and sequences marked in red and bold were obtained in the present study. The scale bar indicates the expected mean number of substitutions per site according to the model of sequence evolution applied. For reasons of clarity, the number of sequences has been reduced to up to 4 sequences per clade. The full phylogenetic tree can be found in Supplementary file 1: Fig. S1.

Phylogenetic analysis of the partial 16S rRNA sequences revealed three distinct clusters (Fig. 4, Supplementary File 1, Fig. S1). One sequence (**PQ834270**) grouped with *Mhc* and *Mhf*. Sequence **PQ834272** formed a separate clade with *Mycoplasma* sp. from a dog in Cambodia, while **PQ834271** clustered with *Mycoplasma* sp. previously isolated from a fox in Slovakia, showing the closest relationship to *C*Mt. The tree topology showed well-supported relationships between the fox-derived sequences and other hemoplasmas from domestic and wild carnivores, with high bootstrap values confirming the robustness of these clusters.

Of the five samples which tested positive in the PCR detecting *Bartonella* spp., 3/5 (60.0 %; 95 % CI 0.147–0.947) showed highest identity to *B. taylorii* (GenBank accession no. **PV170757**), and 2/5 (40.0 %; 95 % CI 0.053–0.853) to *B. rochalimae* (GenBank accession no. **PV170758**), both from Czechia. All sequences showed 100 % identity and 100 % query coverage and were uploaded to GenBank under accession nos. **PX400655**–**PX400659**.

The sample that tested positive in the Trypanosomatidae PCR showed 99.8 % identity and 98 % query coverage to *T. pestanai* in a Eurasian badger (*Meles meles*) from Romania (GenBank accession no. **PP595229**) and was uploaded to GenBank under accession number **PQ826483** (Table 4).

## 4 Discussion

The present study gave us an initial overview of blood-borne pathogens (BBPs) in foxes in Saxony-Anhalt. In this German federal state, extensive studies on BBPs in wild carnivores have so far only been conducted on grey wolves (*Canis lupus*), European wildcats (*Felis silvestris*), and raccoons (Hodžić et al., 2020; Unterköfler et al., 2022, 2024).

In the present study, 277 specimens were examined over a two-year period. With 93.5 % the overall prevalence of pathogens is high, the most frequently detected pathogen being *Hepatozoon* spp. with 76.5 % (Table 3). All sequenced samples were unequivocally identified as *H. canis*, and there are no reports of other *Hepatozoon* species in foxes in Europe. It is therefore highly probable that all positive samples represent *H. canis*. Nevertheless, unsequenced samples are formally designated as *Hepatozoon* spp. to remain conservative in our reporting. For the sake of simplicity, in the following discussion, prevalence and statistical data are generally presented under the designation *H. canis*.

The high prevalence of *H. canis* in the present study aligns with previous European studies, where infection rates in foxes range up to 90 % (Duscher et al., 2014; Ebani et al., 2017; Medkour et al., 2020). Hodžić et al. (2020) found a prevalence of 35 % in grey wolves in Saxony-Anhalt. While this prevalence is lower than that observed in foxes, it is important to note that only 26 wolves were tested by Hodžić et al. (2020), compared to the larger sample size of 277 foxes in the present study. Therefore, differences in sample size make it difficult to directly compare prevalence rates between the two species in this federal state.

Although *Rhipicephalus sanguineus* is considered to be the main vector (Baneth et al., 2001), this tick is not endemic in Northern Germany (Rubel et al., 2022). However, the vector spectrum appears to be broader, as *H. canis* has been detected in various tick species collected from foxes in Germany (Najm et al., 2014b), though vector competence has only been confirmed for few species, such as *Rhipicephalus turanicus* in Italy (Giannelli et al., 2017). Transmission occurs primarily through ingestion of infected vectors (Baneth et al., 2001), but vertical transmission has also been documented (Murata et al., 1993), and transmission through predation is also being discussed (Johnson et al., 2009). Although *H. canis* is generally thought to have low pathogenicity in dogs, with most infections being subclinical or causing mild symptoms (Chisu et al., 2023), it can also be fatal for animals with compromised immune systems or puppies (De Bonis et al., 2021). These findings highlight the role of foxes as potential reservoir hosts for *H. canis*, which could pose a risk of transmission to domestic carnivores. Notably, potentially autochthonous cases of *H. canis* infection in dogs from Germany (Brandenburg) have been reported (Helm et al., 2020), underlining the potential threat to domestic dogs and other carnivores. Future research is needed to better understand the dynamics of parasite exchange between wildlife and domestic animals.

*Babesia* species detected in foxes in Europe are *B. vulpes*, *Babesia canis*, and *Babesia vogeli*, with the latter two being detected only seldomly (Cardoso et al., 2013; Hodžić et al., 2015, 2018; Juwaid et al., 2019; Medkour et al., 2020; Mierzejewska et al., 2021). In contrast, *B. vulpes* is identified regularly in foxes in Europe, with a median prevalence of 31 % (ranging from 1 to 72 %) (Cardoso et al., 2013; Checa et al., 2018; Daskalaki et al., 2018; Dežđek et al., 2010; Duscher et al., 2014; Ebani et al., 2017; Farkas et al., 2015; Hodžić et al., 2015, 2018; Juwaid et al., 2019; Lesiczka et al., 2023; Liesner et al., 2016; Mierzejewska et al., 2021; Millán et al., 2016; Najm et al., 2014a; Sgroi et al., 2021b; Zanet et al., 2014). With 68 %, the occurrence in the present study is high compared both to the European median as well as to German studies (federal states of Brandenburg and Thuringia), where a prevalence of about 46–48 % was described in foxes (Liesner et al., 2016; Najm et al., 2014a).

The vector of *B. vulpes* still remains unknown. While *Ixodes hexagonus* is proposed as the main vector in certain regions (Camacho et al., 2003), the potential of *Ixodes ricinus*, *Ixodes canisuga*, *R. sanguineus*, and *Dermacentor reticularis* as vectors is also debated (Hodžić et al., 2017b; Iori et al., 2010; Lledó et al., 2010; Najm et al., 2014a). Additionally, non-vectorial modes of transmission (i.e. transplacental transmission) are being discussed (Birkenheuer et al., 2010). While dogs usually show severe symptoms when infected with *B. vulpes* (Camacho et al., 2004), the pathogenicity in foxes seems to remain low (Hodžić et al., 2015; Najm et al., 2014a). The high prevalence of *B. vulpes* in foxes strongly suggests their potential to serve as reservoirs for infections in dogs and wild canids, particularly through shared environments and tick exposure. In fact, autochthonous cases of *B. vulpes* infection have also been reported in dogs in Germany (Jetschin et al., 2022; Schäfer et al., 2023; Seibert et al., 2022), further underlining the potential for local transmission.

To the best of the authors' knowledge, the only prior detection of trypanosomes in foxes was reported in Kazakhstan by Galuzo and Novinskaja in 1960 (as cited in Hoare (1972)). The identification of *T. pestanai* in a fox in this study represents the first documented report of trypanosomes in foxes within Europe and the first description of *T. pestanai* in foxes globally. While this kinetoplastid is primarily associated with European badgers (Lindhorst et al., 2024), it has also been reported in a dog in Germany (Dyachenko et al., 2017). The role of badger fleas (*Paraceras melis*) as vectors for *T. pestanai* has been established (Lizundia et al., 2011), and its detection in ixodid ticks was recently reported in Italy (Sgroi et al., 2021a). The detection of *T. pestanai* in foxes during this investigation may reflect behavioral ecology factors, as these canids occasionally share denning sites with badgers (Goszczyński and Wójtowicz, 2001; Kowalczyk et al., 2000). Additionally, Foley et al. (2017) documented *P. melis* infestations in 5 % of Romanian fox populations, suggesting these ectoparasites could facilitate *T. pestanai* transfer between species, emphasizing the necessity for expanded studies to clarify its host specificity and transmission mechanisms.

Hemotropic mycoplasmas have been identified in various wild canids, showing the closest relationships to carnivore-associated hemoplasmas: *Mhc*, *Mycoplasma haemofelis* (*Mhf*), *Candidatus* Mycoplasma haematoparvum (*C*Mhp), *Candidatus* Mycoplasma haematominutum (*C*Hhm), *Candidatus* Mycoplasma turicense (*C*Mt), and uncultured *Mycoplasma* spp. (André et al., 2011; Cabello et al., 2013; Di Cataldo et al., 2020, 2021; Fayos et al., 2024; Koneval et al., 2017; Mascarelli et al., 2015; Millán et al., 2018; Remesar et al., 2025; Santos et al., 2021; Sasaki et al., 2008). However, to the best of our knowledge, only three studies have reported hemoplasmas in foxes, identifying infections with *Mhc* (0.8 %) in Japan (Sasaki et al., 2008), *C*Mt (2 %) in Spain (Millán et al., 2018), and *Mhf*, *Mhc*, and *Mycoplasma* spp. (4 %) in Slovakia (Koneval et al., 2017), the latter showing the closest relationship to *C*Mt, *C*Mhp, and *Mycoplasma* sp. The overall prevalence of 9.7 % in the present study is therefore higher in comparison with the other three studies. In contrast, Unterköfler et al. (2024) found a remarkably high prevalence of *Mycoplasma* sp. among raccoons in Saxony-Anhalt (49 %).

The modes of transmission and potential vectors of carnivore-associated hemoplasmas are not yet fully understood (Fayos et al., 2024; Willi et al., 2007, 2010), although vertical transmission of *Mhc* has been documented in dogs (Lashnits et al., 2019). While the pathogenic potential varies across domestic cats (*Felis catus*) and dogs, depending on the hemoplasma species and host immune status (Willi et al., 2010), limited information is available on clinical symptoms in wild carnivores (Fayos et al., 2024).

The phylogenetic relationships observed in this study (Fig. 4, Supplementary File 1, Fig. S1) suggest that fox-associated hemoplasmas are closely related to strains previously detected in domestic dogs and other wild carnivores, supporting the possibility of cross-species transmission or shared ecological cycles.

Carnivore-related hemoplasmas have also been detected in humans, although these findings remain unpublished (e.g., *C*Mhm in Mexico: Genbank accession no. **OR225697**, **OR225698**, and **OR225699**). This raises concerns that foxes could pose a potential risk to both human and animal health. Further genetic and functional analyses are necessary to assess the pathogenicity of these strains and to clarify their potential zoonotic implications.

The detection of *B. rochalimae* in foxes in this study represents the first detection of this bacterium in Germany. The observed prevalence of 0.7 % falls within the broad range of prevalence rates reported in foxes in European studies (Gerrikagoitia et al., 2012; Greco et al., 2021; Henn et al., 2009a; Hodžić et al., 2018; Millán et al., 2016). However, it must be noted that the prevalences in the aforementioned studies varied highly from each other (0.2 %–100 %). This considerable variability underscores the need for cautious interpretation, as significant differences exist between studies, and sample sizes are very small in some cases. As *B. rochalimae* has also been detected in dogs (Ernst et al., 2020) and even humans (Eremeeva et al., 2007; Traver et al., 2024), foxes could act as reservoirs for this pathogen, potentially contributing to its circulation in wild and domestic animal populations as well as posing a zoonotic risk to humans.

Interestingly, we detected *B. taylorii* for the first time in foxes. Although the pathogen was previously identified in fleas collected from foxes, the authors hypothesized it to originate from a prior blood meal on a rodent host (Víchová et al., 2018). Although this bacterium is primarily associated with rodents (Bown et al., 2004), *B. taylorii*-like species have been occasionally documented in carnivores, with previous reports in Eurasian lynx (*Lynx lynx*) from Czechia (Daněk et al., 2023), raccoons from Canada (Fenton et al., 2021), and stray dogs from Thailand (Bai et al., 2010). To date, its pathogenicity and zoonotic potential remain uncertain (Tahmasebi Ashtiani et al., 2024). Though findings in carnivores may reflect incidental infections, further research is needed to determine its ability to infect non-rodent hosts and assess potential risks for animal and human health. It should also be noted that the detection of *Bartonella* DNA in spleen tissue may not necessarily reflect an active infection of the animal.

Rickettsiales, *Leishmania* spp. and filarioid helminths were not detected among the samples examined. Given that *Rickettsia* spp. and Anaplasmataceae are commonly found in European fox populations (Cardoso et al., 2015; Hodžić et al., 2018; Hofmann-Lehmann et al., 2016), their absence in the samples studied is rather unexpected. Nevertheless, this result should be viewed in light of methodological considerations, as the applied PCR protocols may be less sensitive than nested assays targeting coding gene markers. While L. *infantum* is frequently detected in foxes from Mediterranean regions (Abbate et al., 2019; Cardoso et al., 2015; Davoust et al., 2014; Karayiannis et al., 2015; Risueño et al., 2018; Sgroi et al., 2021b), it has not been reported in foxes in non-endemic Germany (Kaszak et al., 2015). Similarly, filarioid helminths have not been documented in German foxes yet (Härtwig et al., 2016), making their absence unsurprising.

The statistical tests revealed significant variation in the presence of *H. canis* across different districts (*P* = 0.037). The highest prevalence was observed in the district of Wittenberg, the city of Dessau-Roßlau, and Magdeburg (100 %), while the lowest were recorded in the districts of Stendal (0 %) and Jerichower Land (50 %) (Fig. 3A). However, the small sample sizes in these two districts (n = 2) may lead to ambiguous results.

The other significant correlation found was between *Hepatozoon* and the animals’ age (*P* = 0.002, OR Inf, 95 % CI 2.102-Inf) (Table 3). While all juvenile animals tested were infected with *H. canis*, only 74 % of adults showed an infection with this pathogen. In contrast, previous studies did not find a significant relationship between *Hepatozoon* occurrence and the age of foxes (Hodžić et al., 2015, 2018; Najm et al., 2014b; Sgroi et al., 2021b). The high infection rate among juveniles suggests early-life exposure, supporting the theory of vertical transmission (Murata et al., 1993) or early infection (e.g., via vectors inhabiting the fox dens). However, since foxes in this study were classified as juveniles up to the age of one year, other transmission routes remain possible. Further studies focusing on cubs or using more precise age classifications could provide deeper insights.

While this study provides valuable insights into the occurrence of blood-borne pathogens in foxes from Saxony-Anhalt, several limitations should be considered. Only spleen tissue was analyzed, which may have led to an underestimation of pathogens with other tissue preferences or low DNA loads. PCR-based assays detect pathogen DNA but do not indicate active infection or viability. The BTH assay used is not reliably species-specific, which means that unspecific amplification within the Piroplasmida complex cannot be entirely excluded. As only a subset of PCR-positive *Hepatozoon* samples was sequenced, the true species prevalences may differ from the reported estimates. Finally, the lack of clinical or pathological data precludes assessment of disease impact. Despite these constraints, the study provides an important baseline for future research with broader sampling, improved marker resolution, and extended molecular confirmation.

The high prevalence of pathogens such as *H. canis* and *B. vulpes* highlights the role of foxes as potential reservoirs for domestic animals, particularly dogs, through shared environments and vector exposure. In the future, environmental factors such as habitat changes and climate-driven vector shifts are likely to influence pathogen transmission dynamics, emphasizing the need for ongoing monitoring.

## 5 Conclusion

This study highlights the role of foxes as reservoirs for pathogens that may affect domestic animals and humans, potentially contributing to the spread of these pathogens through shared environments and vectors. Future research is needed to better understand transmission dynamics and develop strategies to reduce the risks posed by these pathogens at the wildlife-domestic-human interface.

## CRediT authorship contribution statement

**Zoë Tess Lara Lindhorst:** Writing – original draft, Methodology, Investigation, Data curation. **Manuela Theresa Frangl:** Methodology, Investigation, Data curation. **Barbara Eigner:** Project administration, Methodology, Investigation. **Bita Shahi Barogh:** Project administration, Methodology, Investigation. **Georg Gerhard Duscher:** Methodology, Investigation. **Annette Schliephake:** Methodology. **Wolfgang Gaede:** Methodology, Investigation. **Hans-Peter Fuehrer:** Writing – original draft, Visualization, Validation, Supervision, Resources, Project administration, Methodology, Investigation, Conceptualization. **Mike Heddergott:** Writing – original draft, Supervision, Resources, Methodology, Investigation, Conceptualization.

## Ethics statement

Red foxes are not a protected species in Germany and may be shot by licensed hunters outside the closed season without a special permit. No animals were killed specifically for this study. As the project did not involve live animals or animal experiments, nor did it involve sensitive patient data, the study did not have to be reported to the Ethics and Animal Welfare Committee (ETK) according to point 1.2 and 1.3 of the guidelines regarding Good Scientific Practice (Ethics in Science and Research) of the University of Veterinary Medicine Vienna.

## Funding

This research did not receive any specific grant from funding agencies in the public, commercial, or not-for-profit sectors.

## Declarations of interest

None.

## Figures and Tables

**figs1:**