# Supplementary material for: Molecular analysis of vector-borne pathogens in red foxes (Vulpes vulpes) from Saxony-Anhalt (Germany)
Source: Int J Parasitol Parasites Wildl. 2025 Nov 18;28:101162. doi: 10.1016/j.ijppaw.2025.101162 (PMC12681646; doi:10.1016/j.ijppaw.2025.101162)
Supplement: Multimedia component 1 [file mmc1.docx]

**Supplementary File 1: Table S1.** PCR protocols and cycling conditions used.

| **Target organism (genetic marker)** | **Mastermix protocol** | | | **Cycling Conditions** | | | |
| --- | --- | --- | --- | --- | --- | --- | --- |
|  | **Reagents** | **Unit Reagent** | **Quantity (µl) per tube** | **Temperature (°C)** | **Time** | **Step** | |
| ***Mycoplasma* spp. (16S rRNA)** |  |  |  |  |  |  |  |
|  | H_2_O |  | 12.675 | 94 | 2’ | Initial denaturation |  |
|  | green reaction buffer | 5 x | 5 | 95 | 1' | 40 x |  |
|  | dNTPs | 25 mM | 0.2 | **60** | 1’ |  |  |
|  | TagPolymerase (GoTaq) | 5 u/µl | 0.125 | 72 | 1’ |  |  |
|  | Primer Forward | 10 pmol/µl | 1 | 72 | 7’ | Final extension |  |
|  | Primer Reverse | 10 pmol/µl | 1 | 15 | ~ | Storage |  |
|  | Template | 5 µl | 5 |  |  |  |  |
| **Piroplasmida**  **(18S rRNA)** |  |  |  | **Nest 1** |  |  |  |
|  | H_2_O |  | 30.35 | 94 | 2’ | Initial denaturation |  |
|  | green reaction buffer | 5 x | 10 | 95 | 30’’ | 40 x |  |
|  | dNTPs | 25 mM | 0.4 | **68** | 1’ |  |  |
|  | TagPolymerase (GoTaq) | 5 u/µl | 0.25 | 72 | 1’ |  |  |
|  | Primer Forward | 100 pmol/µl | 2 | 72 | 10’ | Final extension |  |
|  | Primer Reverse | 100 pmol/µl | 2 | 15 | ~ | Storage |  |
|  | Template | 5 µl | 5 | **Nest 2** |  |  |  |
|  |  |  |  | 94 | 2’ | Initial denaturation |  |
|  |  |  |  | 95 | 30’’ | 40 x |  |
|  |  |  |  | **60** | 1’ |  |  |
|  |  |  |  | 72 | 1’ |  |  |
|  |  |  |  | 72 | 10’ | Final extension |  |
|  |  |  |  | 15 | ~ | Storage |  |

**Supplementary File 1: Table S1.** PCR protocols and cycling conditions used (cont.).

| **Target organism (genetic marker)** | **Mastermix protocol** | | | **Cycling Conditions** | | | |
| --- | --- | --- | --- | --- | --- | --- | --- |
|  | **Reagents** | **Unit Reagent** | **Quantity (µl) per tube** | **Temperature (°C)** | **Time** | **Step** | |
| **Trypanosomatida (18S rRNA)** |  |  |  | Nest 1 + 2 |  |  |  |
|  | H_2_O |  | 14.675 | 94 | 5’ | Initial denaturation |  |
|  | green reaction buffer | 5 x | 5 | 94 | 1’ | 35 x |  |
|  | dNTPs | 25 mM | 0.2 | **56** | 1’ |  |  |
|  | TagPolymerase (GoTaq) | 5 u/µl | 0.125 | 72 | 1’ |  |  |
|  | Primer Forward | 10 pmol/µl | 2 | 72 | 5’ | Final extension |  |
|  | Primer Reverse | 10 pmol/µl | 2 | 15 | ~ | Storage |  |
|  | Template | 5 µl | 1 |  |  |  |  |
| ***Bartonella* spp. (*gltA*)** |  |  |  |  |  |  |  |
|  | H_2_O |  | 12.675 | 94 | 5’ | Initial denaturation |  |
|  | green reaction buffer | 5 x | 5 | 94 | 1’ | 40 x |  |
|  | dNTPs | 25 mM | 0.2 | **54** | 1’ |  |  |
|  | TagPolymerase (GoTaq) | 5 u/µl | 0.125 | 72 | 1’ |  |  |
|  | Primer Forward | 20 pmol/µl | 1 | 72 | 10’ | Final extension |  |
|  | Primer Reverse | 20 pmol/µl | 1 | 15 | ~ | Storage |  |
|  | Template | 5 µl | 5 |  |  |  |  |
| ***Rickettsia* spp. (23S-5S rRNA)** |  |  |  |  |  |  |  |
|  | H_2_O |  | 12.675 | 96 | 4’ | Initial denaturation |  |
|  | green reaction buffer | 5 x | 5 | 94 | 1’ | 35 x |  |
|  | dNTPs | 25 mM | 0.2 | **52** | 1’ |  |  |
|  | TagPolymerase (GoTaq) | 5 u/µl | 0.125 | 72 | 2’ |  |  |
|  | Primer Forward | 10 pmol/µl | 1 | 72 | 3’ | Final extension |  |

**Supplementary File 1: Table S1.** PCR protocols and cycling conditions used (cont.).

| **Target organism (genetic marker)** | **Mastermix protocol** | | | **Cycling Conditions** | | | |
| --- | --- | --- | --- | --- | --- | --- | --- |
|  | **Reagents** | **Unit Reagent** | **Quantity (µl) per tube** | **Temperature (°C)** | **Time** | **Step** | |
| ***Rickettsia* spp. (23S-5S rRNA)** |  |  |  |  |  |  |  |
|  | Primer Reverse | 10 pmol/µl | 1 | 15 | ~ | Storage |  |
|  | Template | 5 µl | 5 |  |  |  |  |
| **Anaplasmataceae (16S rRNA)** |  |  |  |  |  |  |  |
|  | H_2_O |  | 11.175 | 95 | 2’ | Initial denaturation |  |
|  | green reaction buffer | 5 x | 5 | 94 | 1’ | 35 x |  |
|  | dNTPs | 25 mM | 0.2 | **54** | 3’’ |  |  |
|  | MgCl_2_ | 25 mM | 1.5 | 72 | 30’’ |  |  |
|  | TagPolymerase (GoTaq) | 5 u/µl | 0.125 | 72 | 5’ | Final extension |  |
|  | Primer Forward | 10 pmol/µl | 1 | 15 | ~ | Storage |  |
|  | Primer Reverse | 10 pmol/µl | 1 |  |  |  |  |
|  | Template | 5 µl | 5 |  |  |  |  |
| **Filarioidea**  **(*COI*)** |  |  |  |  |  |  |  |
|  | H_2_O |  | 10.675 | 94 | 2’ | Initial denaturation |  |
|  | green reaction buffer | 5 x | 5 | 94 | 45'’ | 8 x (red. by 0.5°C each) |  |
|  | dNTPs | 25 mM | 0.2 | **51** | 45’’ |  |  |
|  | TagPolymerase (GoTaq) | 5 u/µl | 0.125 | 72 | 1.5’ |  |  |
|  | Primer Forward | 100 pmol/µl | 2 | 94 | 45'’ | 25 x |  |
|  | Primer Reverse | 100 pmol/µl | 2 | **45** | 45’’ |  |  |
|  | Template | 5 µl | 5 | 72 | 1.5’ |  |  |
|  |  |  |  | 72 | 5’ | Final extension |  |
|  |  |  |  | 15 | ~ | Storage |  |

**Supplementary File 1: Table S1.** PCR protocols and cycling conditions used (cont.).

| **Target organism (genetic marker)** | **Mastermix protocol** | | | **Cycling Conditions** | | |
| --- | --- | --- | --- | --- | --- | --- |
|  | **Reagents** | **Unit Reagent** | **Quantity (µl) per tube** | **Temperature (°C)** | **Time** | **Step** |
| ***Hepatozoon* spp. (18S rRNA)** |  |  |  |  |  |  |
|  | H_2_O |  | 14.675 | 95 | 2’ | Initial denaturation |
|  | green reaction buffer | 5 x | 5 | 95 | 1’ | 35 x |
|  | dNTPs | 25 mM | 0.2 | **58** | 1’ |  |
|  | TagPolymerase (GoTaq) | 5 u/µl | 0.125 | 72 | 1’ |  |
|  | Primer Forward | 100 pmol/µl | 2 | 72 | 7’ | Final extension |
|  | Primer Reverse | 100 pmol/µl | 2 | 15 | ~ | Storage |
|  | Template | 5 µl | 1 |  |  |  |

**Supplementary file 1: Figure S1:** Bayesian Inference (BI) tree featuring 16S rRNA (549 nucleotide positions) sequences of *Mycoplasma* spp. Nodes are marked with Bayesian posterior probabilities and Maximum Likelihood (ML) bootstrap values. Accession number, species name, host, and country are provided for every sequence if available. Sequences written in bold are from *Vulpes vulpes*, and sequences marked in red and bold were obtained in the present study. The scale bar indicates the expected mean number of substitutions per site according to the model of sequence evolution applied.
